# Is Team Resilience More Than the Sum of Its Parts? A Quantitative Study on Emergency Healthcare Teams during the COVID-19 Pandemic

**DOI:** 10.3390/ijerph19126968

**Published:** 2022-06-07

**Authors:** Inge E. M. Hendrikx, Stef C. G. Vermeulen, Vera L. W. Wientjens, Remco S. Mannak

**Affiliations:** 1 Lean Instituut @ Verbeeten, Brugstraat 10, 5042 SB Tilburg, The Netherlands; vermeulen.s@bvi.nl (S.C.G.V.); wientjens.v@bvi.nl (V.L.W.W.); 2 Department of Organization Studies, Tilburg University, P.O. Box 90153, 5000 LE Tilburg, The Netherlands; r.s.mannak@tilburguniversity.edu

**Keywords:** individual resilience, team resilience, COVID-19 pandemic, emergency healthcare, mental health, team familiarity, transformational leadership, self-efficacy, optimism, social support

## Abstract

Due to the COVID-19 pandemic, emergency healthcare workers have come under even more pressure than before, threatening the workers’ mental health and the continuity of care delivered by their teams. This study aims to investigate what conditions increase individual and team resilience, referring to the ability to “bounce back” from stressful situations. We also assess whether team resilience is the sum of the individual resilience of team members, or whether other conditions enhance team resilience and thus continuity of care, despite limited individual resilience. We collected survey data from 129 emergency healthcare team members in the Netherlands to examine to what extent transformational leadership and team familiarity influence the level of team resilience, either directly or mediated by individual resilience, accounting for psychological characteristics and social support. The results show two distinct pathways to enhance team resilience, directly by familiarizing team members with each other and by mobilizing family support, and indirectly but with a much weaker effect, by encouraging team members’ individual resilience through transformational leadership and staffing optimistic team members with high levels of self-efficacy.

## 1. Introduction

The COVID-19 pandemic challenged our healthcare systems tremendously and had major consequences for people’s lives and their mental health [1,2,3]. Emergency healthcare workers directly involved in dealing with the COVID-19 pandemic experienced depression (22.8%), anxiety (23.2%), and insomnia (38.9%) [4,5], threatening the continuity of care delivered by them and their teams [6,7,8,9]. To safeguard employee wellbeing and continuity of care, healthcare organizations should encourage individual and team resilience, referring to the ability to “bounce back” from stressful situations [10,11]. Resilience has been studied as a personality trait, referring to psychological resilience, as a process of structuring feedback and adaptation, and as a capacity that could be developed [11,12,13,14]. From a management science perspective, the capacity view is most suitable for the purpose of this study, because resilience as a capacity can be enhanced with intervention or training in an organizational context [11,15]. In addition to the types of resilience, research defines resilience at the individual, team, and organizational level. In the healthcare hospital context, even on the smallest scale, the care of a patient does not solely rely on one resilient healthcare worker [7]. Teams need to respond and adjust as a unity to overcome the unprecedented challenges the healthcare sector has witnessed and will continue to face [16], indicating the importance of understanding team resilience in addition to individual resilience. Team resilience can be defined as: “the capacity of a team to withstand and overcome stressors in a manner that enables sustained performance; it helps teams handle and bounce back from challenges that can endanger their cohesiveness and performance” [17] (p. 177). A highly resilient emergency healthcare team is able to manage trade-offs between competing goals, collaborate across specialists, and use collective sensemaking [18]. Although recent research suggests that lower-level processes facilitate the development of resilience at higher levels [19,20], the relationship between individual resilience and team resilience is not that obvious. For example, synergetic teams can elevate team resilience above and beyond individual levels. Research is needed to empirically test the interplay between individual and team resilience, as well as their antecedents; for example, to show whether stimuli of individual resilience cumulate in team resilience or whether separate stimuli are required [11,16,19,20].

Literature argues that individual, team-level, and contextual factors play a role in the development of team resilience [11,16,21,22]. This study investigates two main predictors of team resilience, namely the team-level factor, team familiarity, and the contextual factor, transformational leadership, as these conditions are within the circle of influence of an organization to intervene on. First, team familiarity is defined as: “the extent to which team members have personal knowledge of each other’s strengths, weaknesses, preferences, styles’’ [21] (p. 9), thus, as a capacity that could be developed or stimulated, rather than just ‘the duration of work experience’. Team familiarity is associated with individual resilience [23] by creating psychological safety [24], lowering barriers to ask for help [25], and enabling individuals to learn from each other [26,27]. In addition, team familiarity is related to team resilience [21], as team members who are more familiar with each other are more likely to cooperate well by integrating their expertise, resulting in collective action [24,25]. Second, transformational leadership is defined as: “The leader elevates the follower’s level of maturity and ideals as well as concerns for achievement, self-actualization, and the wellbeing of others, the organization, and society” [28] (p. 11). At the individual level, transformational leaders are able to take care of the psychological safety of their team members by creating an environment of openness and trust [24], lowering barriers to ask for help and support, and encouraging feelings of belonging to a team [24,29,30], thereby increasing individual resilience [31]. Transformational leadership is also associated with team resilience [32] by clarifying objectives, showing initiative, and encouraging creative thinking and flexibility to adjust to adversity [33,34]. Both conditions of team familiarity and transformational leadership became salient in the context of emergency healthcare delivery during the COVID-19 pandemic. Many healthcare teams were forced to mix up their team members and reorganize their work responsibilities [35], making unfamiliarity with team members’ expertise inevitable [36]. In addition, team leaders were pressured to simultaneously ensure the psychological safety of their employees [24], convince their team that the crisis situation was surmountable [29], and deal with staff shortages, possibly even taking over care duties themselves [37], requiring them to set priorities.

In sum, previous research has provided arguments for team familiarity effects and transformational leadership effects on either individual resilience [23,31] or team resilience [21,32]. However, there is scant empirical evidence on these effects and their interplay, particularly at times of crises such as the COVID-19 pandemic [16,19]. This study aims to show (I) whether team resilience is a standalone concept, more than the cumulative resilience of team members; (II) whether team familiarity and transformational leadership influence team resilience; and (III) whether these effects are mediated by individual resilience, ensuring team members’ psychological safety, or enhance team resilience directly (synergy effects). The study also accounts for psychological characteristics and social support. Self-efficacy and optimism have been identified as antecedents of individual resilience, helping to adapt to and anticipate different and tense circumstances [31,38]. Moreover, social support from family and friends might facilitate healthcare employees’ ability to bounce back from adversity or stress [39], by providing opportunities to express their emotions and needs and enhancing their psychological safety [24]. Thus, this article aims to investigate to what extent transformational leadership and team familiarity influence the level of team resilience in the context of emergency healthcare teams, and to what extent these relationships are mediated by the level of individual resilience, accounting for psychological characteristics and social support.

## 2. Materials and Methods

### 2.1. Data Collection

This cross-sectional study examines the case of emergency healthcare teams at a clinical teaching hospital with three locations in the Netherlands. This hospital mainly has a regional care-providing function, but also receives patient referrals from other hospitals in the Netherlands because of its leading specialism in areas such as oncology and heart diseases. The Netherlands has a universal healthcare system based on solidarity principles, with equal access for all citizens, enhancing the comparability of hospitals in the Netherlands. The system is managed by the government and supplemented by private insurers and includes three types of hospitals, all privately run: general hospitals, teaching hospitals, and university hospitals. During the COVID-19 pandemic, a central organization was set up to spread out COVID-19 patients over hospitals in order to distribute workload evenly. Despite the spread of patients, hospitals had to postpone ‘regular’ healthcare and focused mainly on COVID-19 patients, making hospitals more comparable to each other during COVID-19 than before the pandemic. Everywhere in the Netherlands, healthcare workers from different departments worked together in emergency healthcare units and teams.

Primary data were collected by distributing a questionnaire through Qualtrics. The data collection took place during the COVID-19 pandemic, namely between July and August 2021. Leaders of 17 emergency healthcare teams distributed the questionnaire in their departments via their newsletters, email, and by laying hard copies in the coffee room of the departments, in a closed box to safeguard the anonymity and confidentiality of the respondents. Employees were asked to fill out the questionnaire on a voluntary basis and were informed that all the identifying data of the employee and hospital would be removed. The respondents were asked to specifically think about their current situation, referring to the COVID-19 period when responding to the questions. A total of 129 respondents adequately completed the survey, excluding one outlier and 20 largely incomplete responses. The 129 respondents in the final sample were divided over 17 different teams, including for example, the coronary care unit, cardiac emergency care, cardiology, ophthalmology, and obstetrics. Table 1 shows the percentage of respondents per function group and per function. Table 1 and the aforementioned details suggest that our sample was largely representative of the larger population of the hospital.

### 2.2. Operationalization and Measurements

The questionnaire was administered in Dutch, implying that the officially validated scales were not used in their original language, but translated from English to Dutch. The researchers and two independent specialists reviewed the initial translation, as this ensures the accuracy of the translation and content validation [40], followed by a pilot test among the intended respondents to avoid content misunderstandings. The official scales are cited in the measurement section below and the Dutch questionnaire is available upon request. All variables investigated in this study are measured as respondent perceptions of latent constructs.

#### 2.2.1. Dependent Variable—Team Resilience

To measure team resilience, the ten-item Campbell-Sills and Stein [41] scale was used based on the Connor–Davidson Resilience Scale [42], as this measurement captures resilience as a capacity, which is central to this study. This study purposely did not use the sum score of individual resilience, but measured team resilience separately from individual resilience. This is in line with the abovementioned expected distinction between the two constructs. Furthermore, five additional items were added to the scale, referring to the context of the COVID-19 pandemic. These questions were conducted by the researcher based on the study of Berg and Aase (2019) [18]. Respondents were asked to indicate on a five-point Likert scale based on agreement (1 = strongly disagree to 5 = strongly agree). Sample items include, for example, “We think of our team as a strong team”, “We can achieve goals despite obstacles”, and “When another COVID-19 wave occurs, our team will work well together to finish all the tasks on time and successfully”. The team resilience scale has been found reliable based on Cronbach’s alpha (α = 0.864).

#### 2.2.2. Mediator—Individual Resilience

The Brief resilience scale (BRS) from Smith et al. [39] was used to measure individual resilience using six items. Respondents were asked to indicate on a five-point Likert scale based on agreement (1 = strongly disagree to 5 = strongly agree). For example, “I tend to bounce back quickly after hard times”. The individual resilience scale has been found reliable based on Cronbach’s alpha (α = 0.892).

An additional principal component analysis with oblimin rotation was used to assess the underlying structure of the combined items of individual and team resilience. The results confirm that all 15 items of team resilience load on component 1, while all 6 items of individual resilience load on component 2, with only one item partially loading on both components. These findings imply that respondents perceive individual resilience and team resilience as two different, and therefore, separable constructs.

#### 2.2.3. Predictors

Transformational leadership. The Global Transformational Leadership scale [GTL] by Carless et al. [43] was used to measure transformational leadership. Respondents were asked to indicate seven items on a five-point Likert scale based on frequency (1 = not at all to 5 = frequently if not always). Respondents were asked for example, “My leader communicates a clear and positive vision of the future”. The transformational leadership scale has been found reliable (α = 0.927).

Team familiarity. Team familiarity was measured by taking the Temporal familiarity measurement scale of Gevers et al. [44], and the scale of Faraj and Sproull [26] using seven items. Respondents were asked to indicate on a five point Likert scale based on agreement (1 = strongly disagree to 5 = strongly agree). For example, “We are familiar with each other’s preferred work pace”. The team familiarity scale has been found reliable based on Cronbach’s alpha (α = 0.766).

Self-efficacy. In order to measure ‘self-efficacy’, six items of the GSE-6 Measurement scale of Romppel et al. [45] were used. This scale is based on the 10-item scale of Schwarzer and Jerusalem [46]. Respondents were asked to indicate on a five-point Likert scale based on agreement (1 = strongly disagree to 5 = strongly agree). For example, “If someone opposes me, I can find the means and ways to get what I want”. The Cronbach alpha has been found reliable (α = 0.703).

Optimism. To measure ‘optimism’, the Life Oriented Test (LOT) from Harland et al. [31] was used. Six items were used containing two dimensions. Respondents were asked to indicate on a five-point Likert scale based on agreement (1 = strongly disagree to 5 = strongly agree). For example, “In uncertain times, I usually expect the best”. The reliability of the optimism scale was moderate based on Cronbach’s alpha (α = 0.630).

Family and friend Support. In order to measure ‘family and friend support’, the multidimensional scale of perceived social support of Ojo et al. [38] was used. A total of six items were used from the ‘family and friend support’ dimension. Respondents were asked to indicate on a five-point Likert scale based on agreement (1 = strongly disagree to 5 = strongly agree). For example, “I get the emotional help and support I need from my family”. When analyzing further, there was a clear distinction between family support (α = 0.912) and friend support (α = 0.924). The principal component analysis showed the two-component model to be more reliable than the one-component model. Therefore, the decision was made to use two components for this predictor. This is in line with the scales used in previous studies [38].

Control variables. Lastly, the control variables, age, tenure, and function, were directly asked in the questionnaire to enhance the internal validity of the research. The continuous variable, age, was measured in seven categories of 10 years, e.g., 20–29 years. Tenure was measured as a continuous variable representing the respondent’s number of years of employment in the team. A total of 14 cases of missing data on the variable ‘tenure’ were replaced with mean scores since analysis with a missing data indicator yielded identical results. The control variable, ‘function’, was divided into three categories, namely, specialists, non-medical staff, and nurses, as shown in the data collection section.

### 2.3. Data Analysis

To get from the data to an empirical answer to the research question, several steps had to be taken. As discussed above, scales were assessed by means of reliability analysis and principal component analysis. In addition, preliminary analyses were conducted, checking for outliers, multicollinearity, normality, linearity, and homoscedasticity. When analyzed, no assumptions were violated, besides the one outlier that was removed, resulting in the final sample size of *n* = 129. Moreover, we used the common latent factor method to test for possible common method bias, which showed no bias. Thereafter, the descriptive statistics, including means, standard deviation, and correlations were calculated to summarize the sample of this study. The conceptual model was tested by means of Structural Equation Modeling (SEM) [47].

## 3. Results

### 3.1. Context Study

The results of our context study show that the COVID-19 pandemic manifested itself within this clinical teaching hospital in extra work hours to deliver the right care, in which employees often worked with different colleagues from different departments. Moreover, employees had different duties, responsibilities, and different team goals. Lastly, on a more personal level, respondents personally felt insecurity around following the right procedures, and the ability to guarantee the right care for all patients. A detailed overview is shown in Table 2, which shows several effects the COVID-19 pandemic has had on the employees in our sample.

### 3.2. Descriptive Statistics and Correlation

First, the descriptive statistics—means, standard deviation and correlations—were calculated to summarize the sample of this study (*n* = 129). The scores for each variable are visualized in Table 3 below. The results show that most respondents rate their team resilience and individual resilience between neutral and positive and that the constructs are positively correlated (r = 0.352; *p* < 0.001). Moreover, the R-square of this correlation is 12.4% (0.352 ^ 2), meaning that individual resilience explains 12.4% of the variance in team resilience, as shown in Table 4. Team resilience is also positively correlated with transformational leadership, team familiarity, self-efficacy, optimism, and family support.

### 3.3. SEM Results

In order to examine to what extent the predictors explain the variance in team resilience, we used Structural Equation Modeling (SEM), with heteroskedasticity-robust standard errors clustered at the team level (17 clusters; *n* = 129). Table 5 shows the Total Effects and Direct Effects; Table 6 shows the Indirect Effects. The Total Effects model shows that the independent variables jointly explain 37.7% of the variance in team resilience (R^2^ = 0.377). Adding individual resilience to the model increases the explained variance by 4.7% (R^2^ Final model = 0.425). This confirms that individual resilience explains part of the variance in team resilience, but team resilience is more than the sum of its parts.

**Figure 1:**
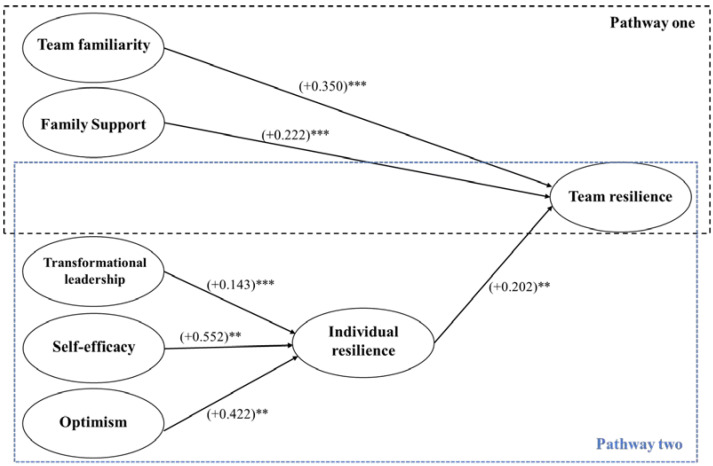
Pathways to team resilience. ** *p* < 0.010; *** *p* < 0.001.

Team familiarity has a positive direct effect on team resilience (b = 0.350; *p* < 0.001), which is not mediated by individual resilience. So, when team members are more familiar with each other, the team as a whole becomes more resilient, without individual members becoming more resilient. Family support also contributes directly to team resilience (b = 0.222; *p* < 0.001) without affecting individual resilience. Transformational leadership has a positive indirect effect on team resilience, which means that transformational leadership stimulates individual resilience (b = 0.143; *p* < 0.001), which in turn enhances team resilience (b = 0.202; *p* < 0.010), but the indirect effect on team resilience is rather weak (b = 0.029; *p* < 0.010). Optimism also has a positive effect on individual resilience (b = 0.422; *p* < 0.010), and an indirect effect on team resilience (b = 0.085; *p* < 0.010). Finally, self-efficacy has a positive effect on individual resilience (b = 0.552; *p* < 0.010), but the indirect effect on team resilience is only marginally significant (b = 0.111; *p* < 0.100). In sum, the study identifies distinct levers for resilience at the individual and team level. Transformational leadership, optimism, and self-efficacy all contribute to individual resilience, but it is team familiarity and family support that helps the team. Figure 1 visualizes these two distinct pathways to encourage team resilience or individual resilience.

To substantiate and interpret the effects and results described above, multiple experts were consulted for in-depth interviews. These experts have backgrounds in the areas of leadership, team development, and continuous improvement within hospital environments, including the hospital chosen for the sample of this study. The results of this study and the additional expert interviews led to valuable theoretical insights and potential underlying mechanisms, but above all have led to practical implications and possible interventions, as are described in the discussion section.

## 4. Discussion

This study empirically investigated whether ‘team resilience is the sum of individual resilience, or more than the sum of its parts’ and how it can be influenced. Previous literature reviews have conceptualized individual and team resilience and identified their conceptual differences [16,19]. However, empirical research, such as this study, is urgently needed to empirically test the conceptualization presented in the aforementioned literature reviews [16,19]. Testing this conceptualization highlights the necessity of interventions at different levels within an organization to enhance both individual and team resilience. Findings show that individual and team resilience are two different constructs. The principal component analysis shows that respondents unconsciously identified the two variables when filling out the questionnaire. This demonstrates that resilience scales validated at the individual level are not necessarily valid to use at the team level, confirming the expectations of recent prior research [13,22]. This implies that studying individual resilience is not sufficient to develop an understanding of resilience at higher levels in the organization; team resilience must be studied in its own right. Moreover, the results show a positive relation between individual and team resilience, suggesting that individuals who have the capacity to react and overcome adversity will create a team environment that positively impacts the resilience of their teams as a whole. However, only 12.4% of the variance in team resilience can be explained by individual resilience, leaving 87.6% of the variance to be explained by other individual, contextual, and team-level factors. This implies that team resilience is a standalone concept that could not be fully explained through individual resilience and that research must identify distinct predictors of team resilience.

When looking at the predictors, this article identifies two distinct pathways to encourage team resilience or individual resilience. On the one hand, familiarizing team members with each other and mobilizing family support stimulates team resilience directly. On the other hand, transformational leadership, and staffing optimistic team members with high levels of self-efficacy stimulates individual resilience, which contributes indirectly but limitedly to the level of team resilience. This is important since team resilience is essential for the continuity of care, while individual resilience is essential for the mental health and wellbeing of healthcare professionals. The findings validate prior conceptual work that distinguished team resilience predictors from individual resilience predictors and called for empirically testing both in a variety of research contexts [16,19]. In the following sections, these two pathways are discussed in more detail.

The first pathway indicates that interventions to enhance team resilience directly should focus on familiarizing team members with each other and mobilizing family support. The results show that team familiarity has a positive significant effect on team resilience but not on individual resilience, meaning that being familiar with other team members does not so much increase one’s own resilience but only that of the team as a whole. The findings correspond to prior research in the context of elite sports [48], but deviate from a study in the healthcare context [23]. The latter study associated individual resilience with a team intervention that enhanced team familiarity and simultaneously provided space for caring and mindfulness. Comparing the findings of both studies leads us to conclude that it must have been the caring and mindfulness elements that contributed to employee psychological safety, while team familiarity only enhances the resilience of the team. A plausible explanation for the latter could be drawn from transactive memory theory. Knowing each other’s strengths, weaknesses, preferences, and styles is a source of synergy, achieving more together than one could possibly have achieved alone [26,49], but it is not a source of mental health and self-protection. On the contrary, team members have a higher level of commitment to collaborate together when they are familiar with each other [44]. The emergency healthcare context is characterized by a lack of slack resources and work that ‘has to be done’. High team familiarity might increase healthcare workers’ tendency to take over work from each other to unburden colleagues. The results demonstrate the importance of differentiating between predictors for individual and team resilience, considering the research context.

Next to team familiarity, the results show that family support has a positive significant effect on team resilience, meaning that the more family support team members perceive, the more resilient the team will be. In contrast to prior literature [39], we did not find a positive family support effect on individual resilience. The reason for these findings could be in the context of this study. The COVID-19 pandemic also highly impacted family life. Hall et al. [7] showed healthcare workers’ primary concerns about their families’ safety in relation to the risks of viral transmission, as well as their high stress attributed to isolation from family and friends. Due to, e.g., quarantine measures, family members might not have been able to deliver the expected mental buffer to express emotions and needs, causing healthcare workers to feel psychologically safe [24]. Instead, family support seems to have manifested as a different type of resource, providing healthcare workers with the ability and support to take over more workshifts flexibly or work more hours (as also shown in Table 2), thus enhancing the team’s capacity to overcome work-demand challenges. This research did not show significant results for friend support, which could be the result of isolation from friends during the COVID-19 pandemic, as a consequence of several lockdowns and social distancing. The results show the importance of developing a contextual understanding of how social support can manifest itself in different forms.

The second pathway to enhance team resilience indirectly is by encouraging individual resilience through transformational leadership, and by staffing optimistic team members with high levels of self-efficacy. In line with prior literature [31], we find a positive, transformational leadership effect on individual resilience, meaning that transformational leaders create a safe and trustful environment which enhances the resilience of their team members [24,30]. Nonetheless, the individual merits of transformational leadership translate into only a weak indirect effect on team resilience. Explanations for the findings may be found in the leaders’ large span of control, which limits their ability to perform leadership tasks or even address the team as a whole [50], and in the great pressure caused by the COVID-19 pandemic, with shortages of staff and beds for COVID-19 patients [37], precluding leaders from their duties to instead help in patient care. “The emergency department has 65 nurses, for which there are four team leaders (working supervisors). On top of that, employees work in 24 h shifts, so it is even possible that they do not physically see their leader for two weeks” (expert interview). Given these challenging circumstances, including quarantine measures that curtail social support from family and friends, transformational leaders seem to have prioritized devoting attention to the mental health and wellbeing of individual team members and creating a psychological safe environment, but leaving team resilience to the team. The findings demonstrate the importance of research on priority setting by leaders in crisis situations.

Furthermore, our results show that optimism and self-efficacy have a positive effect on individual resilience. These findings corroborate previous studies claiming that psychological characteristics such as optimism and self-efficacy can enhance the resilience of individuals [31,38,51], because individuals who are optimistic and express self-efficacy are more likely to feel psychologically safe [24], to be goal oriented, to succeed in handling difficult situations, and to believe in themselves, than those who have less of these characteristics [52,53,54].

In sum, the study demonstrates the importance of examining individual resilience and team resilience as distinct constructs with separate predictors, and the interplay between the two constructs is limited.

### 4.1. Practical Implications

This study reveals several practical implications for managers who, at times of crisis, want to ensure the continuity of care and service delivery and simultaneously strive to safeguard the health and wellbeing of their employees. The findings are acquired in the context of emergency healthcare at the time of the COVID-19 pandemic but may be informative for other situations as well. The ability to bounce back from adversity, either as an individual or as team, are two distinct, loosely coupled capacities. Of course, both individual and team resilience are of importance for well-functioning organizations, but enhancing the former is hardly sufficient to increase the latter, while direct team-level interventions are available. Accordingly, organizations in pursuit of interventions that enhance individual and team resilience could best opt for an intervention portfolio for both the short-term as well as the long-term. The suggestions on interventions for both pathways are described in more detail below.

First, organizations can encourage team resilience directly by enhancing team familiarity and mobilizing family support (pathway one). Organizations could make sure that familiar teams stay together and become increasingly familiar with each other. However, when mixing up teams seems necessary, one should consider investing in time-efficient interventions to familiarize team members with each other in urgent situations with great shortages in staff. Advised interventions, as part of daily operations, are stand-up meetings and hot debriefings [55] and considering team familiarity when forecasting work schedules (balancing stability and flexibility). These examples are time efficient and effective techniques to enhance team familiarity in the context of emergency healthcare. Additionally, interventions can be conducted in ‘off the job’ training, by investing in getting to know each other’s strengths, weaknesses, preferences, and styles.

Second, to enhance team resilience by family support, organizations should think about policies to stimulate and facilitate the work–life balance of healthcare workers. This social support aids employees with the necessary resources to cope with and recover from stressful events [38]. Hospitals have asked much of their healthcare workers. Post pandemic, and with considerable elective healthcare to catch up on, policy makers and organizations should question the pressure they put upon healthcare workers and their work–life balance, and therefore, the support they can receive.

Third, our study shows that transformational leadership, self-efficacy, and optimism help increase individual resilience, which to a limited extent also makes teams more resilient. The findings of pathway two also offer areas of intervention for organizations. Leaders should be aware that a transformational leadership style contributes to the resilience of their team members, and should draw attention to times when challenges have been overcome, and stimulate individuals to communicate with each other and jointly think about how to overcome new challenges. In order to do so, leaders should be visible and ensure that concerns have been heard, and provide support to reduce concerns as much as possible.

Fourth, organizations can also incorporate interventions with regards to employees’ optimism and self-efficacy. Hiring or forming teams with optimistic people with high self-efficacy benefits the organization by ensuring employee mental wellbeing, without denying that employees who have less of these characteristics can be an asset to the organization for many other reasons. It is also advised to be aware of colleagues who lack or have lower degrees of optimism or self-efficacy since these employees might be more prone to stress, and therefore, organizations should pay more attention to these individuals. Indeed, for this latter group of employees in particular, transformational leaders can make a difference.

### 4.2. Limitations and Future Research

Several limitations of this study must be considered. First, data were collected at one hospital in the Netherlands. Although significant results were found and the relative amounts of COVID-19 patients and working methods were comparable in other hospitals during the data collection, future research with larger sample sizes and in different contexts is needed to explore the generalizability of the results. A second limitation concerns the subjective measurements of the variables, as the respondents were asked to assess themselves and their team. Although this approach comes with benefits, such as individuals knowing more about themselves than others might be aware of [56], particularly when it concerns topics such as their own resilience, self-assessment also comes with disadvantages. One potential issue is that of “common method bias”. However, the common latent factor method showed no such bias. Third, although efforts have been made in maximizing the validity of the study in relation to our operationalization and measurement, we adopted validated English questionnaires and translated and administered them in Dutch. We, therefore, did not use earlier validated scales of our variables in the Dutch language. Finally, although we insinuated causality between our variables, a cross-sectional design does not allow us to examine causal direction. Future research using experimental or longitudinal design is recommended to gain more insight on the relationships between the studied constructs.

For future research, we suggest the following directions. First, this study applies a capacity perspective on resilience, rather than resilience as a personality trait or process. Other resilience perspectives also require future research attention. For example, the process perspective of structuring feedback and adaptation [14] offers fruitful avenues for future research, which might result in relevant organizational design interventions for management. Moreover, we specified our conceptual model based on careful literature review and context assessment, but some of the literature does mention other potentially relevant constructs. For example, since the COVID-19 pandemic affected the entire hospital, it could be interesting to study resilience at the organizational level to explore the capacity of a hospital to bounce back and recover from an impactful event. Future research is needed to gain a more comprehensive understanding of individual, team, and organizational resilience as a capacity, process, or trait.

Second, team familiarity was shown to be an important predictor of team resilience but did not necessarily make individuals more resilient. As mentioned earlier, the emergency healthcare context within this study has been characterized by a lack of slack resources, with work that cannot be postponed. It remains unclear if in a context rich in slack resources, team familiarity leads to higher levels of individual resilience. It is recommended to examine under which conditions team familiarity does or does not increase individual and team resilience. Moreover, it is debatable whether extra capacity of new and unknown team members always adds up to benefits for the team, with team familiarity in mind. It is recommended that future research examine whether mixing up teams, or keeping teams together, results in better employee outcomes, including factors such as job satisfaction and absenteeism, and/or better patient outcomes, including factors such as the quality of treatment, survival rates, and satisfaction.

Third, family support seems to have manifested as a different type of resource as expected; not as a buffer for mental health but as a type of support providing individuals with the ability to take over work shifts flexibly or work more hours. Future research could be performed to explore the mechanisms of family support in this context, fulfilling mental support on the one hand, and supporting choices like flexibility in employability on the other hand.

Fourth, due to the exceptional pandemic context and given the role of the (transformational) leader, a fruitful research avenue to explore is whether healthcare workers found mental support at work with their leaders, and to what extent and how this affected resilience. Moreover, as the large span of control of a transformational leader could have been a plausible explanation for the weak indirect effect on team resilience, it is recommended to further examine the effect of the span of control of a leader in relation to the effect on team resilience.

Fifth and finally, this study used the GLT questionnaire consisting of seven transformational leadership behaviors. Future research could explore if certain behaviors have a large influence on team resilience, and create an understanding of why this is the case.

## 5. Conclusions

This study contributes to the current literature on the cross-level interaction between individual and team resilience, showing the unique value of team resilience. Individual resilience does not cumulate one-on-one into team resilience. Instead, individual and team resilience appear to be separate constructs that are partly related, but only explain a limited part of each other’s variance. Moreover, the results of this article show two distinct pathways to enhance team resilience, either directly, by familiarizing team members with each other and by mobilizing family support, or indirectly but with a much weaker effect, by encouraging individual resilience through transformational leadership and staffing optimistic team members with high levels of self-efficacy. More specifically, transformational leadership, self-efficacy, and optimism help the individual, but it is team familiarity and family support that help the team.

## Figures and Tables

**Table 1:** Overview of sample.

Function Group	Function
Nurses (51.9%)	Nurses (51.9%)
Sepcialists (23.3%)	Doctor assistants (11.6%)
	Managers (caregiver 0.8%)
	Paramedics (7.8%)
	Medical specialists (3.1%)
Non-medical (24.8%)	Administrative staff (7.0%)
	Supporting staff (7.0%)
	Managers (10.9%)

**Table 2:** Overview of the effects of COVID-19 pandemic on healthcare employees in our sample.

Extra stress at the workplace	66.9%
Extra work hours	65.9%
Work together with different colleagues from other departments	56.9%
Felt insecure about the ability to guarantee the right care for all patients	53.1%
Felt insecurity around following the right procedures	51.5%
Other duties and responsibilities	50.8%
Other shared goals	37.7%
Less time for personal health	37.7%
Less times for personal development	31.5%

**Table 3:** Descriptive statistics.

	Mean	SD	Min.	Max.
1. Team Resilience	3.739	0.445	2.600	5.000
2. Individual Resilience	3.767	0.630	1.667	5.000
3. Transformational Leadership	3.595	0.672	2.000	5.000
4. Team Familiarity	3.846	0.511	1.429	5.000
5. Self-Efficacy	3.814	0.427	2.500	5.000
6. Optimism	3.752	0.462	2.333	4.667
7. Family Support	4.222	0.572	2.667	5.000
8. Friend Support	4.269	0.628	2.667	5.000
9. Tenure	10.678	9.994	0.000	40.000
10. Age	4.000	1.358	2.000	6.000
11. Specialist	0.233	0.424	0.000	1.000
12. Non-Medical Staff	0.248	0.434	0.000	1.000

**Table 4:** Correlations.

	1	2	3	4	5	6	7	8	9	10	11
1	-										
2	0.352 ***	-									
3	0.362 ***	0.299 ***	-								
4	0.498 ***	0.166 ^†^	0.354 ***	-							
5	0.197 *	0.545 ***	0.269 **	0.206 *	-						
6	0.247 **	0.476 ***	0.184 *	0.152 ^†^	0.398 ***	-					
7	0.259 **	0.092	0.121	0.105	0.068	0.276 **	-				
8	0.092	0.181 *	0.125	0.129	0.180 *	0.271 **	0.531 ***	-			
9	0.004	0.056	−0.028	−0.039	−0.017	0.023	0.032	−0.009	-		
10	0.130	0.172 ^†^	0.066	−0.003	0.085	0.075	0.027	−0.015	0.487 ***	-	
11	0.101	−0.132	−0.062	0.089	−0.176 *	0.004	−0.075	−0.021	0.005	−0.041	-
12	0.095	0.132	0.233 **	−0.013	0.258 **	0.121	−0.067	0.021	−0.066	0.133	−0.316 ***

^†^ *p* < 0.100; * *p* < 0.050; ** *p* < 0.010; *** *p* < 0.001.

**Table 5:** Structural Equation Model (SEM).

	Total Effects		Direct Effects		Direct Effects	
	DV: Team Resilience		M: Individual Resilience		DV: Team Resilience	
	B	SE	B	SE	B	SE
Constant	0.949	(0.712)	−0.435	(0.481)	1.037	(0.694)
Individual Resilience					0.202 **	(0.065)
Transformational Leadership	0.106 ^†^	(0.055)	0.143 ***	(0.033)	0.077	(0.056)
Team Familiarity	0.349 ***	(0.063)	−0.005	(0.076)	0.350 ***	(0.050)
Self-Efficacy	0.034	(0.081)	0.552 **	(0.174)	−0.077	(0.091)
Optimism	0.090	(0.074)	0.422 **	(0.140)	0.005	(0.068)
Family Support	0.203 ***	(0.053)	−0.092	(0.130)	0.222 ***	(0.053)
Friend Support	−0.104	(0.066)	0.058	(0.120)	−0.116 *	(0.058)
Tenure	0.037	(0.033)	0.055	(0.039)	0.026	(0.032)
Age	−0.002	(0.004)	0.000	(0.004)	−0.002	(0.004)
Specialist	0.135	(0.088)	−0.127	(0.103)	0.161 ^†^	(0.082)
Non-Medical Staff	0.089	(0.097)	−0.127	(0.092)	0.115	(0.092)
R^2^	0.377		0.419		0.425	

^†^ *p* < 0.100; * *p* < 0.050; ** *p* < 0.010; *** *p* < 0.001. DV = dependent variable, M = mediator.

**Table 6:** Structural Equation Model (SEM): Indirect effects.

	Indirect Effects	
	Via: Individual Resilience	
	DV: Team Resilience	
	B	SE
Transformational Leadership	0.029 **	(0.011)
Team Familiarity	−0.001	(0.016)
Self-Efficacy	0.111 ^†^	(0.059)
Optimism	0.085 **	(0.031)
Family Support	−0.018	(0.028)
Friend Support	0.012	(0.026)

^†^ *p* < 0.100; ** *p* < 0.010. DV = dependent variable.

## Data Availability

The data are not publicly available to ensure confidentiality.
